# “What’s measured gets done”: a call for a European semester for cancer to improve cancer outcomes in Central and Southeastern Europe

**DOI:** 10.1186/s13690-023-01165-5

**Published:** 2023-08-08

**Authors:** Christoph Zielinski, Christiane Thallinger, Alexander Rödiger

**Affiliations:** 1 Central European Cooperative Oncology Group (CECOG), Ohmanngasse 26, HQ Vienna, 1190 Austria; 2 Department of Internal Medicine I, Medical University Vienna, 18-20 Waehringer Guertel, 1090 Vienna, Austria; 3 MSD International Business GmbH, Kriens, Switzerland

**Keywords:** Oncology, Mortality, Inequality, Central and South-Eastern Europe (CEE)

## Abstract

Cancer mortality varies widely across Europe, and survival depends on where you live. In particular, the inequality between countries in Central and South-Eastern Europe (CEE) and Western Europe (WE) is striking. The COVID-19 pandemic has brought existing inequalities into sharp focus, and the economic disruption it has caused threatens to deepen them. The Central European Cooperative Oncology Group (CECOG) has created a platform with the aim to reduce health inequalities and to improve patient access to cancer care. The subject of discussion is the value of new treatments to create willingness to invest in improving cancer outcomes while managing the budget. The platform includes various stakeholders as scientific leaders, policy makers, payers, patients and industry.

## Main text

As recently noted, in the US cancer death rate continued to decline by 1.5% from 2019 to 2020, contributing to a 33% overall reduction since 1991 and an estimated 3.8 million deaths averted [1]. This progress increasingly reflects advances in prevention, diagnosis, and treatment. In parallel, in Europe but also in Asia Pacific or the US Cancer Control Plans had been launched to address cancer comprehensively and strategically [2, 3]. Given the positive US statistics and the widely recognized prioritization of cancer – will we able to beat cancer in the near future?

Despite the positive news of declining cancer mortality rates future progress may be attenuated by rising incidence for breast, prostate, and uterine corpus cancers [1]. Due to demographic change and population growth cancer continues to become disease burden number one [4]. Finally, as with health in general but more nuanced in cancer due to its life-threatening impact, inequalities in cancer care across the world and within regions including Europe are concerning. The 5-year survival rate for colorectal cancer is nearly 20% lower in Croatia than in Belgium. Eastern European countries rank also lower in screening, and access to healthcare resources is limited or at least delayed [5, 6]. Europe’s Beating Cancer Plan recognizes these inequalities, and their reduction has become a key priority with the flagship initiative of an Inequalities Registry.

Local stakeholder involvement is critical for the success of policies addressing inequalities. Between 2018 and 2020 a multi-stakeholder group (CECOG GOIA group*) under the leadership of the Central European Cooperative Oncology Group (CECOG) convened a series of conferences in Vienna, Bucharest, Zagreb and Warsaw, with a three-fold objective.

1. to reduce systemic health inequalities and improve patient access and funding to cancer care;
2. to focus on prevention, screening, early diagnosis, access to state-of-the-art cancer diagnosis and treatment;
3. to propose solutions which ensure progress in the implementation of cancer control plans.

The purpose of the initiative, funded by CECOG, AstraZeneca and MSD, was to create a platform for stakeholder dialogue in the above-mentioned countries and to make recommendations for Europe’s Beating Cancer Plan. The outcome was the so-called CECOG Cancer Dashboard which identifies key metrics for Eastern European countries and shall contribute to the “Inequalities Registry” in the context of the European Commission’s “Europe’s Beating Cancer Plan” [7].

This document includes prevention (e.g. tobacco control, screening, HPV vaccination), diagnostics (e.g. molecular testing, radiologic interventions, workforce), treatments (e.g. time to access), clinical research (e.g. clinical trials) and involvement of patient advocacy groups (e.g. definition of patient pathways). The dashboard is based on two publications of the CECOG initiative which analyze the current state of cancer care in Central and Eastern Europe in the EU and provide recommendations for change [6, 8].

Another result of the CECOG exchange meetings was the view that the impact of cancer policies on cancer patients’ lives depends on two critical factors. First, cancer control policies must be aligned with and integrated in public health strategies, particularly those addressing prevention related to disease prevention. Between 30 and 50% of all cancer cases are preventable [9]. Prevention offers the most cost-effective long-term strategy for the control of cancer. In addition, screening programs should be universally available, implemented and attract as much persons as possible. A report on actual and optimal radiotherapy capacity in 33 European countries described large availability deficiencies of equipment, primarily teletherapy units. Lack of qualified human resources for optimal delivery of radiotherapy services exacerbates the problem [6].With regard to treatment, there has been an unprecedented wave of innovations in cancer treatment in the past few years. These developments may have a considerable budget impact and require new pathways and financing mechanisms to ensure timely patient access [10]. Finally, better political, and societal awareness about cancer is needed: due to demographics and lifestyle change cancer is already in some countries disease burden number one and will soon become that in several other European countries.

Second, besides integration in public health strategies cancer policies are successful if they are guided by objectives, create transparency and are driven by consensus-based actions. The Cancer Inequalities Registry of Europe’s Beating Cancer Plan will provide data points to inform activities [11], which is a necessary but not sufficient step. Another insight of the CECOG process was that additional mechanisms are needed to enable implementation and progress. This includes local consensus-building and local stakeholder such as healthcare professionals, patients, payers etc.

The so-called European Semester process may serve as a role model or may integrate health outcomes targets including cancer [12]. This European framework provides integrated surveillance and coordination of economic and employment policies across the European Union. Since its introduction in 2011, it has become a forum for discussing EU countries’ fiscal, economic and employment policy challenges under a common annual timeline.

A European Semester process for cancer would not only map the status quo but also ensure progress in cancer outcomes:

1. it calls on stakeholders to agree on key indicators and targets;
2. it requires to create and collect the data and to measure progress; and
3. it triggers improvement at local level.

A simple example may illustrate the last point: although nearly all Eastern European countries have National Cancer Control Plans (NCCP) in place, differences still exist compared to other European countries because of a lack of implementation and measuring progress. A recent study showed that breast cancer screening coverage is still lowest in Eastern European countries (49%). If the maximum of full coverage was reached, 23% of breast cancer deaths could be prevented in Eastern countries, two times as much as Northern countries [13].

Europe’s Beating Cancer Plan, launched in 2021, represents a unique new opportunity for Central and Eastern European countries, considering the challenge cancer will be for future societies in Europe [11]. The mid-term in 2023, when the first horizontal report will be issued [14], represents an opportunity to review what enables implementation and to develop a process which ensure long-term progress in reducing inequalities in cancer outcomes.

## Data Availability

The GOIA dashboard can be found on the CECOG homepage: https://www.cecog.org/goia/fighting-cancer-the-cecog-cancer-dashboard-for-cee/.

## Abbreviations

CEE: (Central and South-Eastern Europe)
CECOG: (Central European Cooperative Oncology Group)
